# Superior efficacy of Adalimumab in treating childhood refractory chronic uveitis when used as first biologic

**DOI:** 10.1186/1546-0096-9-S1-P220

**Published:** 2011-09-14

**Authors:** G Simonini, A Taddio, M Cattalini, R Caputo, C DeLibero, I Pagnini, S Naviglio, L Lepore, R Cimaz

**Affiliations:** 1 Anna Meyer Children’s Hospital and University of Florence, Florence, Italy; 2 Institute of Child Health, IRCCS Burlo Garofolo, University of Trieste,Trieste, Italy; 3 Pediatric Clinic, University of Brescia, Brescia, Italy

## Background

We previously reported that Adalimumab is more efficacious than Infliximab in maintaining remission of chronic childhood uveitis.

## Aim

To compare the efficacy of Adalimumab when used as first anti-TNFα therapy *versus* Adalimumab used after the failure of a previous anti-TNFα (Infliximab). Open-label, comparative, multi-centre, cohort study of childhood non-infectious chronic uveitis.

## Methods

26 patients (14 F, 12 M; median age: 8.6 years) with refractory, vision threatening, non-infectious active uveitis were enrolled. Due to the refractory course of uveitis to previous DMARD treatment, Group 1 received Adalimumab (24 mg/sq mt, every 2 weeks), as *first* anti-TNFα choice; Group 2 received Adalimumab, as *second* anti-TNFα drug, due to the loss of efficacy of Infliximab, after a period of at least 1 year (5 mg/kg at weeks 0, 2, 6 and then every 6–8 weeks). Both groups received Adalimumab for at least 1 year of treatment. Primary outcome was, once remission was achieved, the time to a first relapse. Time to achieve remission, and time to systemic corticosteroid discontinuation were also considered.

## Results

**Figure 1:**
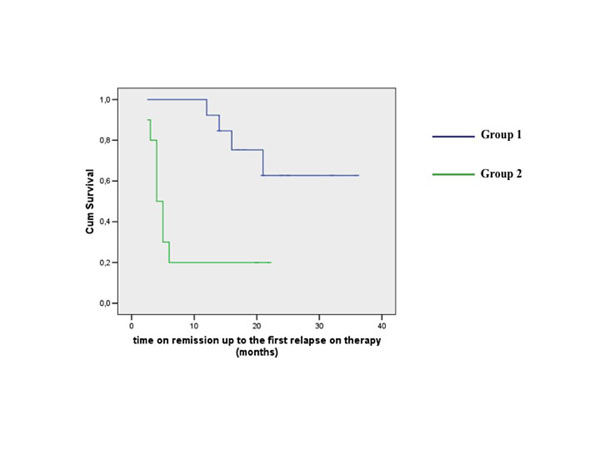


14 children (10 with JIA, 3 with idiopathic uveitis, 1 with Behçet’s disease) were recruited in Group 1; 12 children (7 with JIA, 3 with idiopathic uveitis, 1 with early-onset sarcoidosis, 1 with Behçet’s disease) in Group 2. Cox-regression analysis did not show statistical significant differences between the two groups with regard to time to achieve remission, whilst Group 2 needed a longer time to discontinuation steroid (median ±SE: 7 ±1.7 *vs* 3 ±0.9 months, CI 95%: 3.6-10.4 *vs* 1.1-4.8, p<0.001) and a lower probability to steroid discontinuation during the first 12 months of treatment (Mantel-Cox χ2 4.12, p<0.041). In long-term follow-up, Group 1 had higher probability of uveitis remission (time to first flare) than Group 2 during the time of treatment on Adalimumab (median ±SE: 18 ±1.1 *vs* 4 ±0.6 months, CI 95%: 15.6-27.5 *vs* 2.7-5.2, Mantel-Cox χ2 10.1, p<0.002) (Figure 1).

## Conclusions

Even if limited to a relatively small group, our study suggests a better efficacy of Adalimumab when used as *first* anti-TNFα treatment in chronic childhood uveitis.

